# Aconitum Napellus in Its Relation to the Female Sexual System

**Published:** 1881-12

**Authors:** C. Carleton Smith

**Affiliations:** Philadelphia


### ACONITUM NAPELLUS IN ITS RELATION TO THE FEMALE SEXUAL SYSTEM.

C. Carleton Smith, M. D., Philadelphia.

In all cases where we find the peculiar mental symptoms so characteristic of this drug, we have in it a curative agent which acts with the utmost promptness.

Even in chronic cases (so-called) it acts curatively, provided the symptoms calling for its exhibition are present, and this, notwithstanding the statement made by many authors that *Aconite* is never to be thought of except in strictly acute cases.

In suppression of the menses from getting the feet wet, or by *sudden checking* of the *perspiration*, this remedy must be thought of first: Young girls for various reasons will sometimes foolishly plunge their feet in cold water to stop the monthly flow, and as a consequence, severe congestive headache supervenes, with fever, restlessness and great excitability, followed later by epistaxis and palpitation of the heart. In such cases *Aconite* is the remedy. Should the patient be of a plethoric habit the drug will act all the more promptly.

Another sphere for the action of *Aconite* is in suppression of menses from the effects of *fright.* In such instances this remedy will be found invaluable, provided we have the mental symptoms so peculiar to it, and also the *dizziness* on rising up from a reclining position, and the red face changing to a pale, colorless hue.

In these suppressions, either from fright or from exposure to cold winds, young girls will often seek our advice under *great alarm.*

They *fear* that the flow will never be re-established, and that in consequence they will, sooner or later, become the victims of Phthisis Pul. Or they are *fearful* and full of tribulation, lest their friends will give them the credit of being in the family way. *Aconite* is the remedy here, and a few doses in a high potency will be generally all-sufficient to restore the menstrual function, and with its appearance dissipate also the mental symptoms.

Womb.—*Aconite* gives us stitching pains in the right side of fundus; they are sharp and shooting. *Kali carb.* has *stitches* in all portions of the womb, and in the Kali patient we find the menstrual flow is so *acrid* as to cause the inner portion of the thighs to be covered with an eruption: With these pains we are likely to have great sensitiveness to touch of the abdominal walls.

*Helonias* has nipples very sensitive, cannot bear the clothing to touch.

*Aconite* also gives us pain like labor in the uterus; these are of a *pressing* nature, causing patient to *bend double* like *Colocynth.*, but this position while it relieves the *Colocynth.* patient does *not relieve* the *Aconite* patient.

Ovaries.—Inflammation of these organs from checked perspiration, or from sudden checking of the menstrual flow from fright, or riding in cold winds, or from getting wet when overheated, with fear on the part of the patient that she will not recover, accompanied with bitter vomiting and cold sweat, *Aconite* promptly administered will bring complete relief.

Vagina.—Walls of vagina become very *dry* and *hot* and of course, very *sensitive* to the least impression. *Helonias* has *hot burning* vagina also, but instead of *dryness* there appears a curdy deposit like apthæ. The *Helonias* patient is not excited like the *Aconite* patient, but, on the contrary, is languid and weak

In pregnancy when labor pains are exceedingly *rapid* and *violent*, overwhelming the patient, face and sometimes the whole body bathed in a hot, steamy perspiration, patient screaming out with anguish, says she will surely die, face red, eyes glistening, thirst inordinate, breath short, but withal no progress in labor, *Aconite* will quiet the storm.

When the first milk is being secreted after parturition and the patient is very feverish and delirious also, breasts *hard*, *hot*, milk scanty, *Aconite*, one dose will bring such marked relief that it is scarcely ever necessary to repeat it. During labor, piano music is unbearable to the *Aconite* patient, and she cries out against it vehemently.

This drug is especially applicable in persons having dark hair and eyes, and in plethoric persons who lead indolent lives.

*Arnica* follows admirably after *Aconite*, and *Sulphur* antidotes the abuse of the drug.

There is a wide difference between the mental symptoms of *Aconite* and those of *Helonias*. The former is all excitement; the latter apathetic, languid, prostrated.

